# Malassezia May Contribute to Basal Cell Carcinoma Development via Inflammatory and Oxidative Stress Pathways

**DOI:** 10.1002/mbo3.70401

**Published:** 2026-09-08

**Authors:** Xiaolei Zhu, Wen Zhang, Tinghong Ye, Heng Zhang, Jufang Tan, Yi Sun

**Affiliations:** ^1^ Department of Dermatology, Hubei Provincial Clinical Research Center for Diagnosis and Therapeutics of Pathogenic Fungal Infection Jingzhou Hospital Affiliated to Yangtze University Jingzhou Hubei Province China; ^2^ Department of Pharmacy, Hubei Provincial Clinical Research Center for Diagnosis and Therapeutics of Pathogenic Fungal Infection Jingzhou Hospital Affiliated to Yangtze University Jingzhou Hubei Province China; ^3^ Department of Neonatology, Hubei Provincial Clinical Research Center for Diagnosis and Therapeutics of Pathogenic Fungal Infection Jingzhou Hospital Affiliated to Yangtze University Jingzhou Hubei Province China

**Keywords:** basal cell carcinoma, cell proliferation, inflammatory factors, Malassezia, metagenomic sequencing, oxidative stress

## Abstract

Basal cell carcinoma (BCC) is the most common malignant skin tumor. Skin‐resident lipophilic *Malassezia* yeasts are associated with various cutaneous disorders, while their correlative patterns and potential biological effects in BCC tissues remain insufficiently defined. We used RT‐qPCR screening of archived FFPE BCC specimens and metagenomic sequencing of three paired fresh tumor and peritumoral tissues to characterize tissue‐associated *Malassezia* colonization. *M. globosa* was the most abundant species in FFPE samples and was also detectable in fresh tissues. In vitro functional assays (CCK‐8, EdU) in HaCaT keratinocytes and A‐431 epidermoid carcinoma cells showed that 12 h stimulation with optimal concentrations of *M. globosa* (1.2 × 10^7^ CFU/mL) and *M. yamatoensis* (1.6 × 10^7^ CFU/mL) significantly promoted epithelial cell proliferation. Transcriptome sequencing and subsequent RT‐qPCR validation further showed that both strains significantly upregulate pro‐inflammatory genes (*IL‐1β*, *IL‐6*, *TNF‐α*) and oxidative stress‐related genes (*SOD1, SOD2)* in these cell lines. Collectively, our findings describe a correlative association between *Malassezia* colonization and BCC lesions and offer preliminary in vitro mechanistic clues.

## Graphical abstract

*Malassezia* (predominantly *M. globosa*) colonizes BCC tissues. In vitro fungal stimulation accelerates epithelial proliferation and modulates inflammatory/oxidative stress cascades potentially relevant to BCC biology.

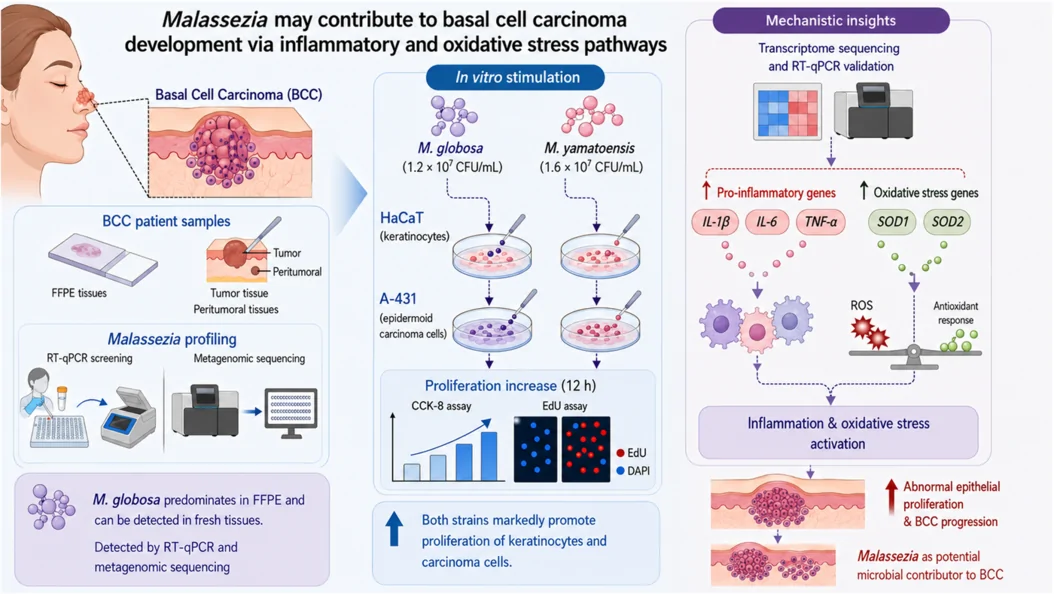

**Abbreviations**
BCC: basal cell carcinoma
CCK‐8: cell counting Kit‐8
CT: cancer tissues
EdU: 5‐Ethynyl‐2′‐deoxyuridine
PBS: phosphate‐buffered saline
PT: peritumoral tissues
RT‐qPCR: real‐time quantitative PCR
SCC: squamous cell carcinoma
SOD: superoxide dismutase
UV: ultraviolet

## 1 Introduction

Basal cell carcinoma (BCC) is the most common form of skin malignancy, with recent statistics reporting more than 5 million new cases diagnosed worldwide each year. This rising incidence poses a serious public health challenge and has gained significant attention across multiple sectors of society (Hu et al. 2022). Although BCC generally grows slowly, its locally invasive nature can cause deep tissue necrosis (Tan et al. 2023). In severe cases, the tumor extends into soft tissue, cartilage, and bone, leading to ulceration, functional impairment, and even irreversible disfigurement, exerting a profound negative impact on patients' quality of life (Stundys et al. 2024). Furthermore, the high prevalence and complex treatment of BCC contribute to a considerable economic burden on healthcare systems. Ultraviolet (UV) radiation is recognized as the primary risk factor for BCC (Leiter et al. 2020). However, in recent years, research focus has shifted toward the interaction between tumors and the microbiota, particularly the skin‐resident fungus *Malassezia*.

*Malassezia*, a genus of lipophilic yeasts that naturally inhabit human skin, has long been associated with dermatological conditions such as seborrheic dermatitis, pityriasis versicolor, and atopic dermatitis (Hall and Noverr 2017). Emerging evidence further connects this fungal genus to systemic disorders, including Crohn's disease, breast cancer, colorectal cancer, and pancreatic cancer (Limon et al. 2019; Liu et al. 2024; Rathie et al. 2023). Collectively, these studies suggest that *Malassezia* may exert functional effects in both cutaneous and systemic pathological processes.

Several biological properties of *Malassezia* are hypothesized to facilitate malignant progression. These include epithelial barrier disruption, upregulation of pro‐inflammatory cytokines, and degradation of the extracellular matrix (ECM), which are recognized processes associated with tumor initiation and malignant transformation (Yang et al. 2022). Both inflammation and oxidative stress are key drivers of tumorigenesis and cancer development, as they promote cellular proliferation while inhibiting apoptosis through multiple molecular pathways (Sorriento 2024). The interplay between *Malassezia* and inflammatory signaling pathways therefore offers a promising direction to unravel tumorigenic mechanisms.

Although *Malassezia* is well‐studied in benign skin disorders and systemic diseases, its functional mechanistic linkage to BCC remains poorly characterized. Earlier works have preliminarily explored the potential oncogenic relevance of *Malassezia* to skin malignancies: a 2011 hypothesis paper proposed that *Malassezia* may drive skin carcinogenesis via aryl‐hydrocarbon receptor ligand secretion (Gaitanis et al. 2011), while a 2024 molecular investigation identified and classified *Malassezia* species within human BCC lesions, documenting fungal colonization in tumor tissues (Koç Yıldırım et al. 2024). Nevertheless, these studies merely remain at the level of correlative observation without in‐depth mechanistic exploration. The present study combines RT‐qPCR detection of archived FFPE blocks and metagenomic mycobiome sequencing of paired fresh tumor‐peritumoral tissues with in vitro epithelial cell models to characterize the proliferative effect of *Malassezia* and explore related inflammatory and oxidative stress cascades. Our findings identify a correlative association between *Malassezia* colonization and BCC and provide a basis for follow‐up in vivo research.

## 2 Methods

### 2.1 Research Specimen

Paraffin‐embedded tissue samples from 50 patients with BCC were retrieved from the Department of Pathology, Jingzhou Hospital affiliated with Yangtze University, from 2023 to 2024. Moreover, three patients with biopsy‐confirmed BCC who underwent surgical treatment from September 2023 to September 2024 at the outpatient and inpatient departments of the same hospital were enrolled. During surgery, tumor tissue and adjacent normal tissue samples (at least 5 mm from the tumor margin, with a minimum size of 5 mm) were collected. Immediately after excision, samples were placed in phosphate‐buffered saline (PBS) and transferred to a −80°C freezer within 30 min for subsequent experiments. The study protocol adhered to the principles outlined in the Declaration of Helsinki and was approved by the Ethics Committee of Jingzhou Hospital affiliated with Yangtze University (Approval number: 2024‐187‐01). After fully explaining the research purpose, process, potential risks and benefits to all the participants involved in the study, they all signed a written informed consent form before being enrolled in the study.

Inclusion criteria were: (1) Patients with a preliminary clinical diagnosis of BCC; and (2) Patients who had undergone histopathological examination of skin tissue.

Exclusion criteria were: (1) Subjects who cannot tolerate local surgical excision; (2) active superficial fungal infection at or near the lesion site (including seborrheic dermatitis and pityriasis versicolor), assessed by the treating dermatologist during preoperative evaluation; (3) topical antifungal use within 2 weeks or systemic antifungal use within 4 weeks prior to tissue collection, as documented in the medical record; and (4) known immunosuppressive conditions (organ transplantation, HIV/AIDS, ongoing immunosuppressive therapy) or genetic syndromes predisposing to BCC (e.g. Gorlin syndrome, xeroderma pigmentosum).

Lesions were classified by anatomical site into the following mutually exclusive categories: nose, periocular (eyelids and canthi), other facial sites (cheek, forehead, chin, and temple), ears, scalp, neck, trunk, and extremities.

### 2.2 Sample Assay and Sequencing Analysis

Tumor tissue (CT) and peritumoral tissues (PT) were collected from three pairs of BCC patients. All fresh tissue specimens were preserved on dry ice and sent to Beijing Biomarker Technologies Corporation for metagenomic high‐throughput sequencing. All DNA extraction, host DNA depletion, library preparation, and bioinformatic analyses described below were completed by the sequencing contractor following their standardized pipeline. The workflow included: (1) DNA extraction and quality control: Genomic DNA was isolated from fresh tissues with a commercial extraction kit, followed by concentration and integrity assessment to verify eligibility for subsequent processing. (2) Host DNA depletion, microbial DNA enrichment, and library construction: Given the extremely low fungal biomass and overwhelming human host DNA background typical of skin tissues, the sequencing provider performed bead‐based size selection to deplete large human genomic fragments and retain microbial DNA, followed by microbial DNA concentration via vacuum centrifugation. The enriched microbial DNA was then subjected to fragmentation, end repair, A‐tailing, adapter ligation, and PCR amplification according to standard Illumina (3) Raw data filtering: Raw reads were filtered to remove low‐quality sequences, adapter dimers, and residual reads mapped to the human reference genome (GRCh38). (4) Metagenomic: De novo assembly of the filtered clean sequences was performed using MEGAHIT, excluding sequences < 200 bp from further analysis; (5) Gene prediction and abundance analysis: where gene prediction was performed using MetaGeneMark. Non‐redundant gene catalogs were constructed using CD‐HIT and their abundance was quantified using Salmon. No extraction blanks or no‐input library negative controls were included in this metagenomic workflow.

### 2.3 Microbial Diversity Analysis in Cancer and Peritumoral Tissues

β‐diversity was evaluated using principal component analysis (PCA). QIIME and R software were used to perform PCA and visualize distributional characteristics between CT and PT samples.

### 2.4 Cell Line Sources and Culture Conditions

The human immortalized keratinocyte cell line HaCaT (Catalog No. CP‐H113) and human epidermoid carcinoma A‐431 (Catalog No. CL‐0015) were obtained from Wuhan Supeno Life Technology Co. Ltd. (China). *M. globosa* (Catalog No. B319469) and *M. yamatoensis* (Catalog No. B222570) were purchased from Ningbo Mingzhou Biotechnology Co. Ltd. (China). A‐431 epidermoid carcinoma cells are not a bona fide BCC cell line. We utilized this keratinocyte‐derived epidermoid carcinoma cell as an in vitro surrogate model to investigate *Malassezia*‐mediated epithelial proliferation, inflammatory response and oxidative stress responses. This cell model shares multiple core signaling cascades relevant to cutaneous malignancy with BCC, yet it lacks the unique subtype‐specific molecular signatures of authentic BCC lesions.

To activate *Malassezia* strains, a loopful of the fungal mycelium from slant cultures was inoculated into Leeming‐Notman liquid medium and incubated at 37°C with shaking for 24–36 h to generate suspensions. The activated suspensions were spread on Leeming‐Notman agar plates and incubated at 37°C for 7 days to obtain viable colonies.

### 2.5 Selection Rationale for *Malassezia* Species In Vitro Experiments

*M. globosa* was selected for in vitro experiments because it was the predominant *Malassezia* species detected in paraffin‐embedded BCC tissue at a relative abundance of 15% (Figure 4), and it is well recognized as one of the most abundant *Malassezia* species colonizing human skin. *M. yamatoensis* was included as a second test species (Figure 4) to evaluate whether the observed biological effects are specific to *M. globosa* or shared among different *Malassezia* species.

### 2.6 Reagents and Equipment

*Malassezia* culture medium was obtained from Qingdao Jingteng Biotechnology Co. Ltd. (China). Cell Counting Kit‐8 (CCK‐8) was purchased from Beijing Solarbio Science & Technology Co. Ltd. High‐glucose DMEM medium, fetal bovine serum (FBS), 0.25% trypsin, and EdU Cell Proliferation Kit were purchased from Yisheng Biotechnology Co. Ltd. *Malassezia*‐specific primers were designed and synthesized by Sangon Biotech Co. Ltd. (Wuhan). A NanoDrop One spectrophotometer was purchased from Thermo Fisher Scientific (China). A real‐time quantitative PCR (RT‐qPCR) system was obtained from Suzhou Yari Biotechnology Co. Ltd. A CO_2_ incubator was purchased from Haier Bio‐Medical, and a microplate reader from Hangzhou Aosheng Instrument Co. Ltd.

### 2.7 DNA Extraction

Genomic DNA was extracted from paraffin‐embedded tissue samples using the Fungal DNA Kit (Yisheng Biotechnology Co. Ltd.) according to the manufacturer's protocol. Paraffin‐embedded tissue sections were deparaffinized before lysis. DNA quality and concentration were assessed using a NanoDrop spectrophotometer, with A260/280 ratios ranging from 1.8 to 2.0, indicating acceptable purity.

Strict contamination prevention measures were implemented during FFPE dewaxing, DNA extraction, and pre‐PCR preparation. All pre‐amplification operations were performed in an independent laminar flow hood with 30 min UV disinfection before and after each experiment. All filter pipette tips and experimental reagents were autoclaved prior to use to eliminate exogenous microbial contaminants. Disposable gloves were worn throughout all experimental procedures; all stock reaction reagents were aliquoted into single‐use portions to avoid contamination induced by repeated freezing and thawing. Pre‐PCR reagent preparation and post‐PCR product detection were physically separated in distinct laboratory zones to block aerosol cross‐contamination. DNA was stored at −20°C until further analysis.

### 2.8 RT‐qPCR From Paraffin‐Embedded Tissue

Primers were synthesized by Sangon Biotech Co. Ltd. (Wuhan). The species‐specific primers targeting the internal transcribed spacer (ITS) region of the ribosomal RNA gene cluster were designed based on published sequences (Xie et al. 2014). The primer sequences are listed in Table 1. The qPCR reaction system (20 μL total volume) consisted of: 10 μL 2× SYBR qPCR Master Mix, 1 μL forward primer, 1 μL reverse primer, 6 μL nuclease‐free water, and 1 μL DNA template. The amplification protocol initial denaturation at 95°C for 15 min, followed by 40 cycles of denaturation at 95°C for 10 s, annealing at 60°C for 20 s, and extension at 72°C for 20 s. All reactions were performed in triplicate.

**Table 1:** ITS region‐based species‐specific primer sequences for RT‐qPCR detection of Malassezia species (Xie et al. 2014).

Species	F/R	Premier sequence(5′−3′)
*Malassezia*	*Malassezia*‐F	GTAGACTCCATCTAAAGCTAAAT
	*Malassezia*‐R	CTTTTAACTCTCTTTCCAAAGT
*M. furfur*	*M. furfur*‐F	GTGAATTGCAGAATTCCGTGAAT
	*M. furfur*‐R	GAGCCTGTTTCTTGCGAAACA
*M. globosa*	*M. globosa*‐F	GTGAATTGCAGAATTCCGTGAAT
	*M. globosa*‐R	GAGCTTTTTCTAGAGAAGAAAAG
*M. obtusa*	*M. obtusa*‐F	GTGAATTGCAGAATTCCGTGAAT
	*M. obtusa*‐R	GCGAGCCTGTTTAGCAAGAAA
*M. pachydermatis*	*M. pachydermatis*‐F	GTGAATTGCAGAATTCCGTGAAT
	*M. pachydermatis*‐R	GAGCCTGTAGTTTCCCACAG
*M.restricta*	*M. restricta*‐F	GTGAATTGCAGAATTCCGTGAAT
	*M. restricta*‐R	GCGAGCCTGTGCTAGGTA
*M. slooffiae*	*M. slooffiae*‐F	GTGAATTGCAGAATTCCGTGAAT
	*M. slooffiae*‐R	CTTTTCGAGCGAGCCTACCAA
*M. sympodialis*	*M. sympodialis*‐F	GTGAATTGCAGAATTCCGTGAAT
	*M. sympodialis*‐R	TACAATCCCCAGGCAGCAA
M. *yamatoensis*	*M. yamatoensis*‐F	GTGAATTGCAGAATTCCGTGAAT
	*M. yamatoensis*‐R	GCCAGCCTCGCAAGGCAT
M. *Japonica*	*M. japonica‐*F	GTGAATTGCAGAATTCCGTGAAT
	*M. japonica‐*R	TGTACGAGACACTGGCAGGCA
M. *dermatis*	*M. dermatis‐*F	GTGAATTGCAGAATTCCGTGAAT
	*M. dermatis‐*R	GTTTCCCAGGCAGCGGCA

*Note:* F, forward primer; R, reverse primer.

To identify potential contamination within the amplification system, no‐template negative controls were included in every batch of qPCR assays, in which nuclease‐free water replaced the DNA template while all other reaction components remained identical to sample reactions.

### 2.9 Cell Culture

HaCaT and A‐431 epidermoid carcinoma cell lines were cultured in DMEM supplemented with 10% FBS and 1% penicillin‐streptomycin. Cells were maintained at 37°C in a humidified incubator with 5% CO_2_. When cell confluence reached 80%–90%, they were subcultured and used for subsequent experiments.

### 2.10 Assessment of Cell Metabolic Activity and Proliferation

CCK‐8 Assay*:* HaCaT and A‐431 epidermoid carcinoma cells in the logarithmic growth phase were adjusted to 1 × 10^5^ cells/mL and seeded into 96‐well plates at 100 μL per well. After adherence, the culture medium was replaced with medium containing different concentrations of *Malassezia*, and cells were treated for 6 , 12 , and 24 h. Subsequently, 10 μL CCK‐8 reagent was added to each well and incubated for 1 h. Absorbance was measured at 450 nm with a microplate reader, and the proliferation rate was calculated.

EdU Assay: Cells in logarithmic growth phase were seeded into 12‐well plates and allowed to adhere. The culture medium was then replaced with medium containing the optimal concentration of *Malassezia*, and cells were treated for 6 , 12 , and 24 h. Following treatment, cells were incubated with EdU medium, fixed, stained, and washed according to the kit protocol. Fluorescence microscopy was used to evaluate proliferation; red fluorescence indicated active DNA replication in cells.

### 2.11 RNA Sample Preparation, Library Construction, and Sequencing

HaCaT and A‐431 epidermoid carcinoma cells were stimulated with *M. globosa* and *M. yamatoensis* for 12 h before RNA extraction and sequencing. Total RNA quality was assessed before library preparation. Clean reads were aligned to the reference genome using HISAT2 to ensure accurate and efficient mapping of sequence data. Transcriptome assembly was performed with StringTie to reconstruct transcripts for downstream analysis. Differentially expressed genes (DEGs) were identified using a threshold of |fold change | ≥ 1.5 and false discovery rate (FDR) < 0.05. Functional interpretation of DEGs was conducted via KEGG pathway enrichment analysis. All sequencing and bioinformatics procedures were performed by Biomaker Biotechnology Co. Ltd. (Qingdao, China). Three biological replicates were analyzed per sample.

### 2.12 RNA Extraction

Total RNA was extracted using the Total RNA Extraction Kit (Yisheng Biotechnology Co. Ltd.) according to the manufacturer's instructions. To eliminate genomic DNA contamination, RNA samples were treated with DNase I (RNase‐free).

### 2.13 RT‐qPCR Detection of Inflammatory and Oxidative Stress Factors

To validate functional enrichment analysis, five DEGs from significantly enriched pathways were selected for RT‐qPCR verification, with *β‐actin* as the internal reference gene. Complementary DNA (cDNA) was synthesized, and RT‐qPCR was performed using the SYBR Green One‐Step qRT‐PCR Kit (Yisheng Biotechnology Co. Ltd.). Each reaction (20 μL total volume) contained 10 μL SYBR Green Master Mix, 2 μL cDNA, 1 μL forward primer, 1 μL reverse primer, and 6 μL nuclease‐free water. The amplification conditions were as follows: initial denaturation at 95°C for 10 min; 45 cycles of denaturation at 95°C for 15 s, annealing at 60°C for 1 min, and a final extension at 72°C for 10 min. Relative transcription levels were calculated using the 2^‐ΔΔCt^ method (Livak and Schmittgen 2001). Primers were designed and synthesized by Sangon Biotech Co. Ltd. (Shanghai), and the sequences are listed in Table 2. Each sample was tested in triplicate.

**Table 2:** RT‐qPCR detection of genes and primer sequences.

Gene	F/R	Primer sequence(5′−3′)
*β‐actin*	*β‐actin*‐F *β‐actin*‐R	CCTGGGCATGGAGTCCTGTG TCTTCATTGTGCTGGGTGCC
*IL‐1β*	*IL‐1β*‐F *IL‐1β*‐R	GTGGCAATGAGGATGACTTGTTC TTGCTGTAGTGGTCGGAG
*IL‐6*	*IL‐6* ‐F *IL‐6* ‐R	GACAGCCACTCACCTCTTCA TGAAGAGGTGAGTGGCTGTC
*TNF‐α*	*TNF‐α* ‐F *TNF‐α* ‐R	GTCAGATCATCTTCTCGAACC TTCACCAGGCAAGTCTCCTC
*SOD*_*1*_	*SOD*_*1*_ ‐F *SOD*_*1*_ ‐R	ATGACTTGGGCAAAGGTGGAAATG TTACACCACAAGCCAAACGACTTC
	*SOD*_*2*_ ‐F	TCACCGAGGAGAAGTACCAGGAG
*SOD*_*2*_	*SOD*_*2*_ ‐R	CCGTTAGGGCTGAGGTTTGTCC

*Note:* β‐actin.beta‐actin; IL‐1β, interleukin 1 beta; IL‐6. interleukin 6; TNF‐α, tumor necrosis factor α; *SOD*_*1*_, Superoxide Dismutase 1; *SOD*_*2*_, Superoxide Dismutase 2; F, forward primer; R, reverse primer.

### 2.14 Statistical Analysis

All data are expressed as mean ± standard deviation (SD). Statistical analyses were performed using SPSS 22.0 software, and figures were generated using GraphPad Prism 9. Clinical variables were compared by the chi‐square test, and disease duration was analyzed using the rank‐sum test. All cellular functional measurements of HaCaT and A‐431 cells were analyzed independently via two‐way ANOVA with treatment group and time point as two fixed factors, followed by Tukey's post‐hoc multiple comparison test. A *p* < 0.05 was considered statistically significant, while *p* < 0.01 indicated highly significant differences. All experiments were independently repeated three times to ensure reproducibility.

## 3 Results

### 3.1 Gender and Age Distribution

**Figure 1:**
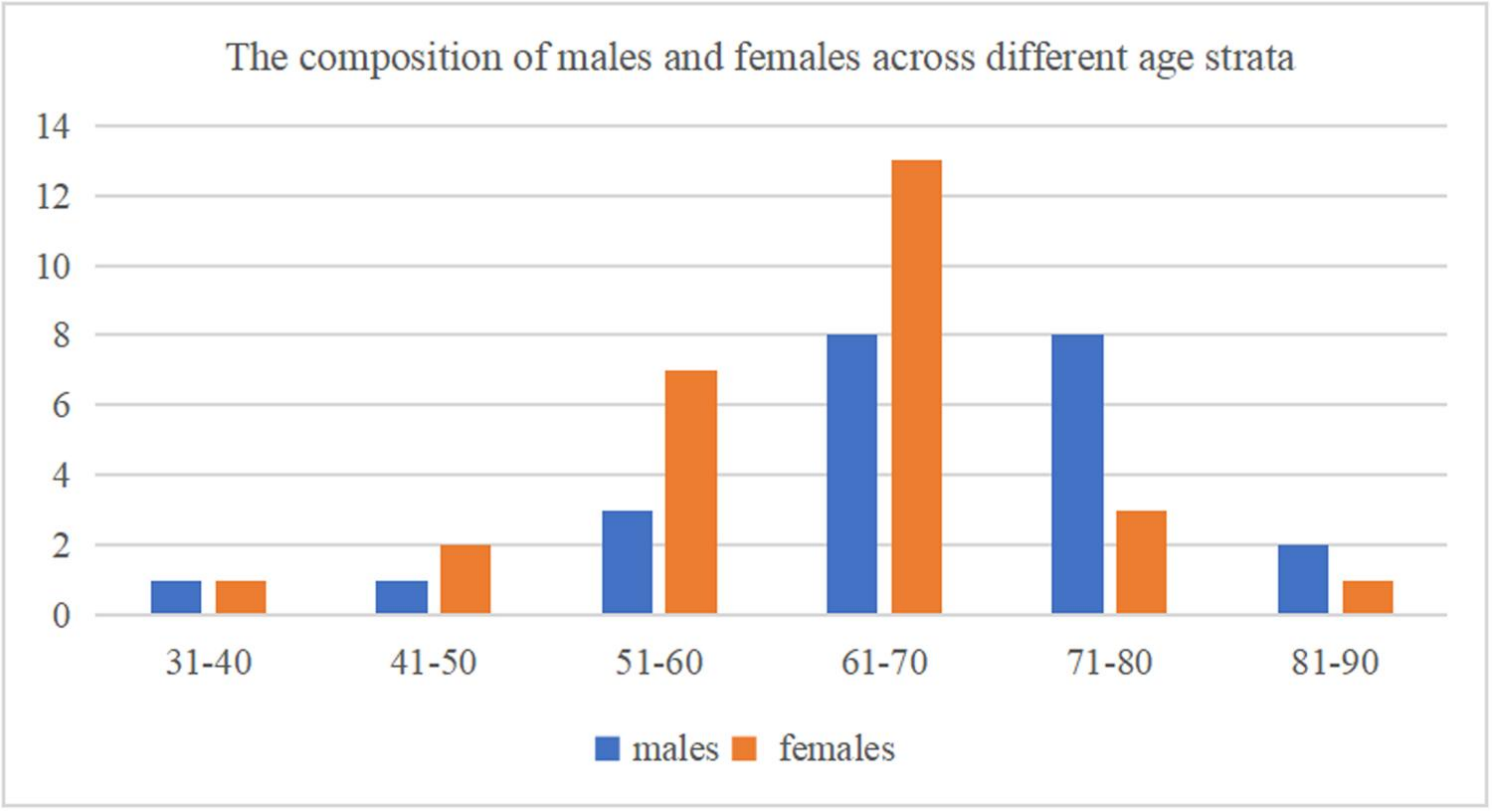
The composition of male and female across different age strata.

Among the 50 patients diagnosed with BCC, the male‐to‐female ratio was 0.85:1 (23 males and 27 females). Patient ages ranged from 34 to 90 years, with a mean age of 66.7 years. Patients categorized into age groups by decade from 30 to 90 years: 2 patients (4%) were aged 31–40 years, 3 patients (6%) aged 41–50 years, 10 patients (20%) aged 51–60 years, 21 patients (42%) aged 61–70 years, 11 patients (22%) aged 71–80 years, and 3 patients (6%) aged 81–90 years. The 61–70‐year age group had the highest incidence, while the lowest was in the 31–40‐year group. The distribution of male and female patients across age groups is shown in Figure 1. Chi‐square test analysis (Table 3) revealed no statistically significant difference in gender composition (*p* > 0.05). Although females appeared to constitute a slightly higher share of participants, the overall case distribution may be comparable between males and females.

**Table 3:** Correlation of sex and age composition in BCC patients (*n* = 50).

Clinical characteristics			Sex			
	Age group	Total	male	female	χ^2^	*p*
Age	30–40	2	1	1	5.23	0.44
	41–50	3	1	2		
	51–60	10	3	7		
	61–70	21	8	13		
	71–80	11	8	3		
	81–90	3	2	1		
		50	23	27		

### 3.2 Gender and Course of Disease

The disease duration was defined as the time interval from initial detection of skin lesions to histopathological confirmation of BCC through surgery or biopsy. Among the 50 patients, the disease duration ranged from 3 months to 54 years. The median duration for these 50 patients was 3 years overall, 5.5 years for males, and 3 years for females. Rank‐sum test analysis showed no statistically significant difference in disease duration between genders.

### 3.3 Lesion Distribution

**Figure 2:**
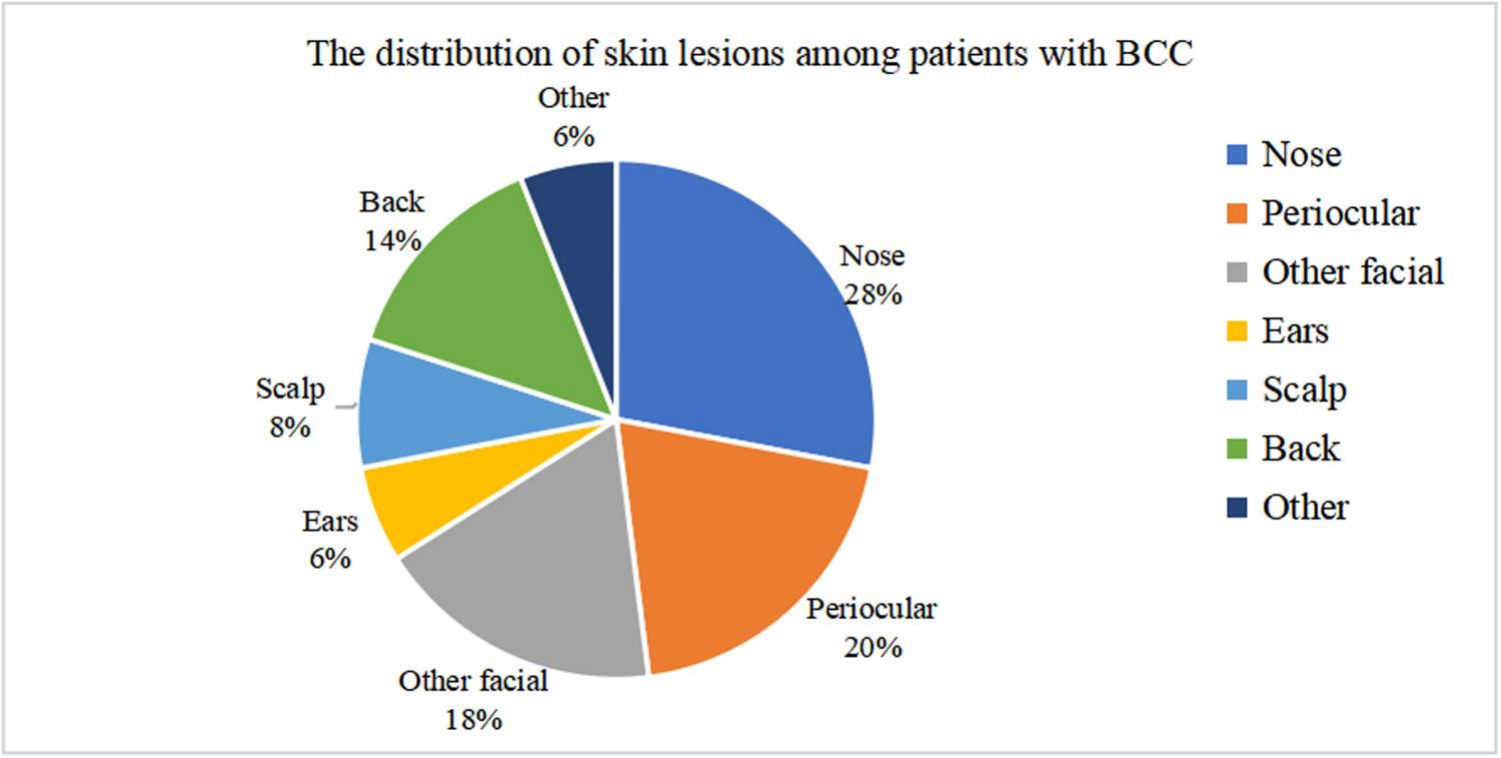
Distribution of basal cell carcinoma lesions in the study cohort.

The anatomical distribution of BCC lesions in our cohort is presented in Figure 2. All head and neck lesions were subdivided into independent anatomical subunits: nasal (14 cases, 28%), periocular (10 cases, 20%), other facial sites (9 cases, 18%), scalp (4 cases, 8%), and ears (3 cases, 6%). Lesions outside the head and neck included the back (7 cases, 14%), with the remaining three cases categorized as other sites (3 cases, 6%).

### 3.4 Histological Subtypes

**Figure 3:**
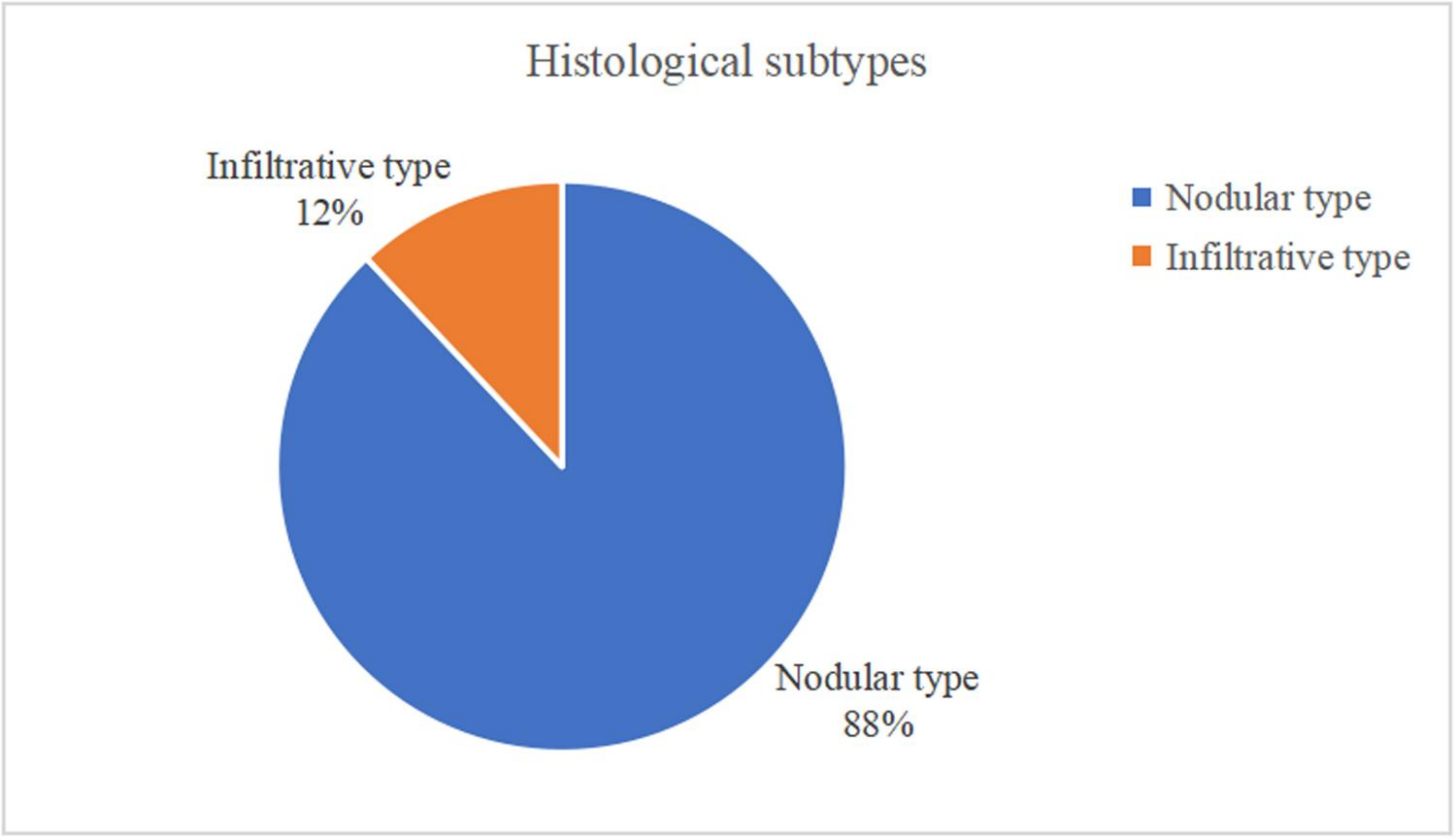
Histological subtypes of BCC in the local patient cohort.

As shown in Figure 3, nodular BCC was the predominant histological subtype among the 50 patients, accounting for 44 cases (88%). Non‐nodular lesions were relatively less common, with the infiltrative type observed in 6 cases (12%). Nodular lesions were primarily distributed on the head and face.

### 3.5 Species Distribution of *Malassezia* in Paraffin‐Embedded Tissue Samples

**Figure 4:**
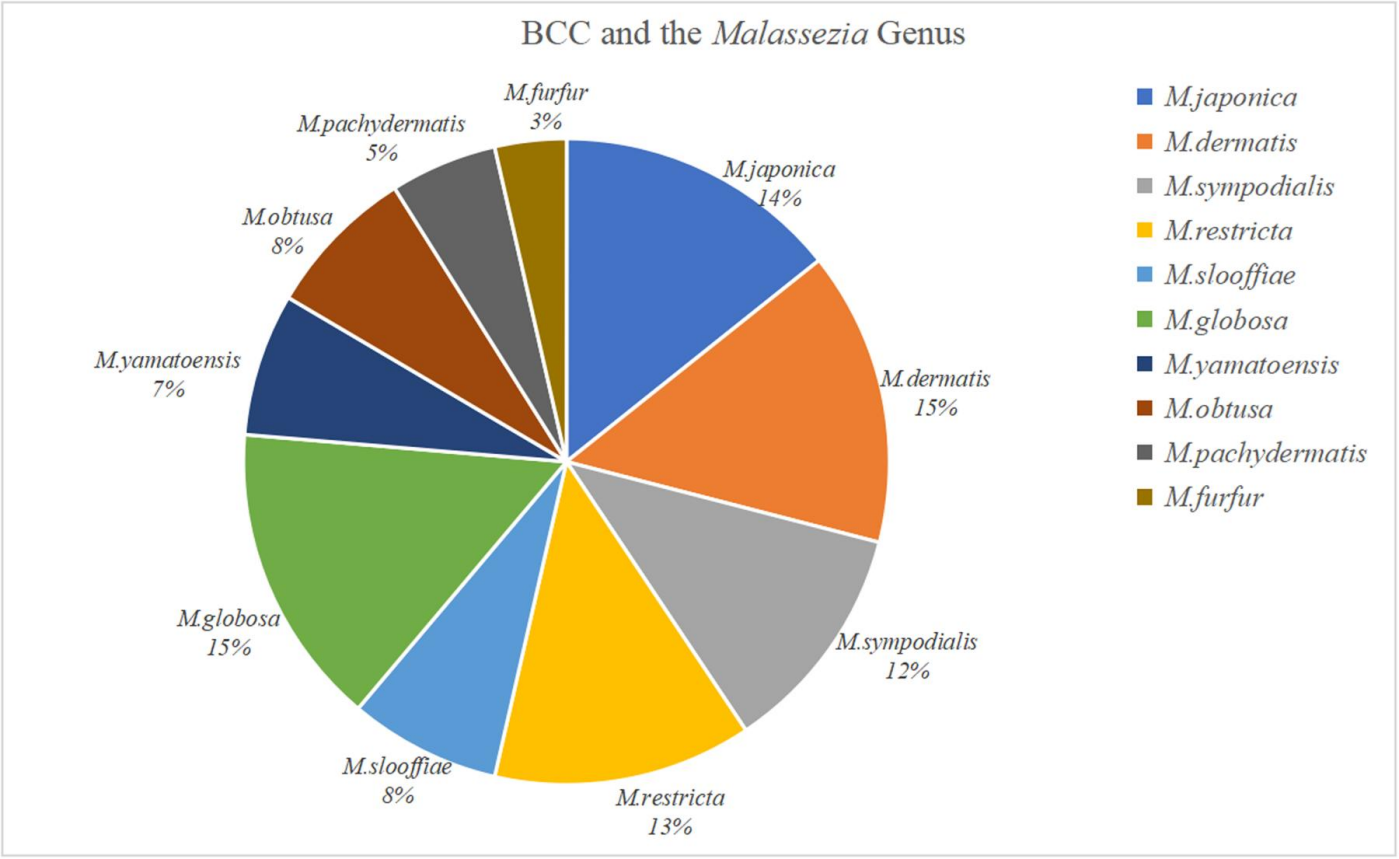
*Malassezia* species detected in patients with BCC.

Figure 4 illustrates the species composition of *Malassezia* in FFPE BCC specimens, where *M. globosa* represented the most abundant taxon at a relative abundance of 15%.

### 3.6 Methodological Approach for *Malassezia* Detection

**Figure 5:**
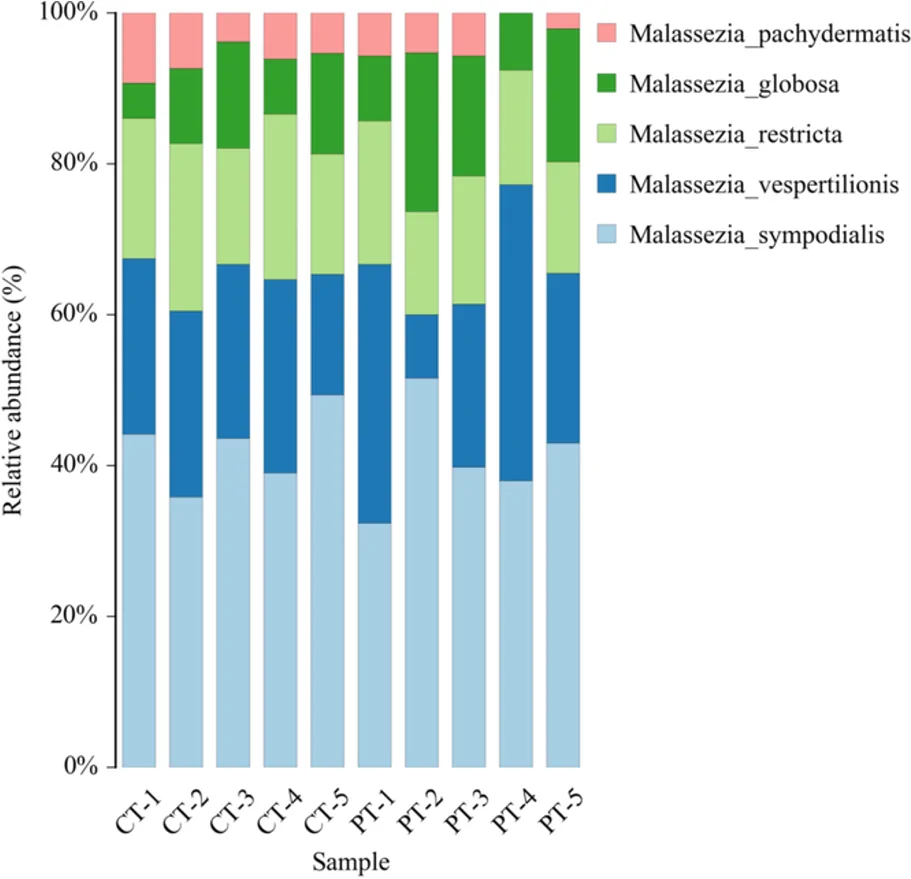
Relative abundance of *Malassezia* species in BCC and peritumoral tissues.

Two complementary approaches were employed to preliminarily characterize *Malassezia* colonization in BCC. First, species‐specific RT‐qPCR was performed on 50 paraffin‐embedded archival tissue samples to assess the presence and diversity of *Malassezia* species (Figure 4). Second, metagenomic sequencing was conducted on fresh‐frozen paired CT and PT samples from only three patients to preliminarily explore species‐level abundance and functional potential (Figure 5). This dual‐method strategy enabled both broad screening and preliminary exploration of the fungal mycobiome in BCC.

### 3.7 β‐Diversity Analysis of *Malassezia* in Cancerous and Peritumoral Tissues

**Figure 6:**
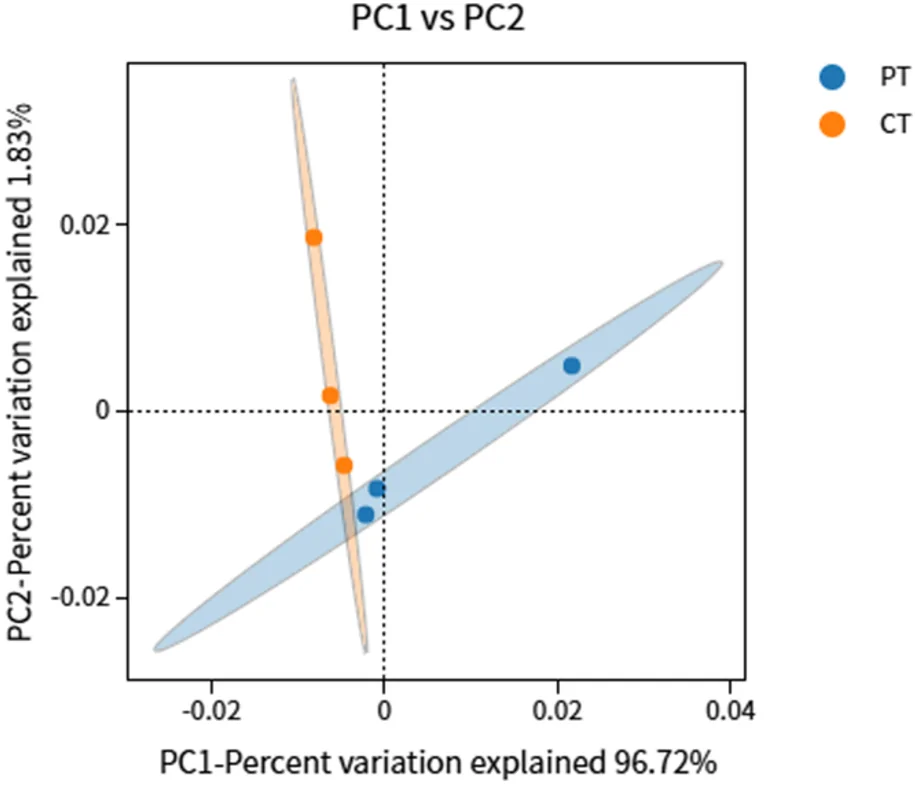
PCA of *Malassezia* communities in BCC CT and PT.

PCA showed separation of fungal communities between cancer tissues (CT, orange) and peritumoral tissues (PT, blue). PC1 and PC2 explained 96.72% and 1.83% of the total variance, respectively, together accounting for 98.55% (Figure 6).

### 3.8 Comparison of the Fungal Genera Abundance Between CT and PT

**Figure 7:**
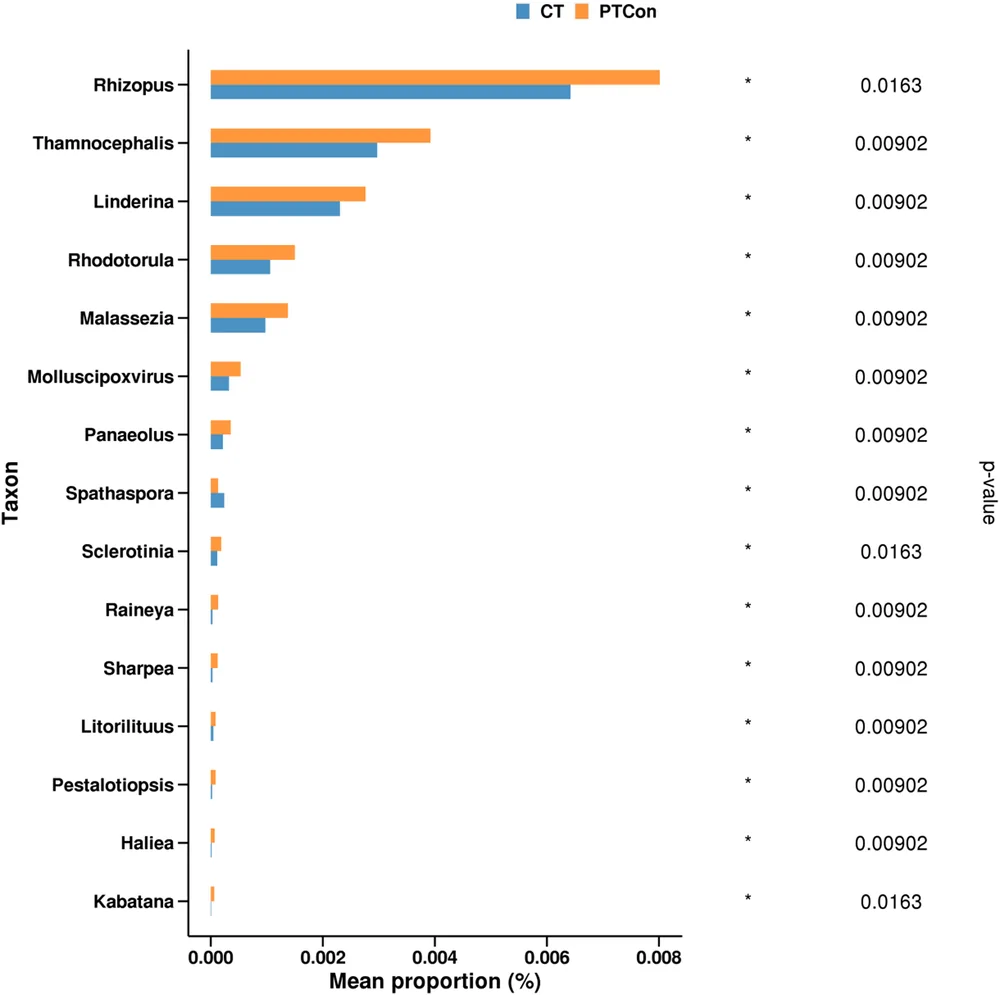
Abundance of *Malassezia* and other differentially abundant taxa in BCC CT versus PT Con. *p < 0.05.

The Wilcoxon rank‐sum test was employed to assess differential fungal abundance between CT and PT (Figure 7). At the genus level, *Malassezia* was shown to be significantly more abundant in PT samples than in CT (*P* < 0.01). Given the small paired‐sample size, this differential abundance is exploratory and warrants confirmation in larger cohorts.

### 3.9 KEGG Pathway Functional Annotation of Differential Microbiota

**Figure 8:**
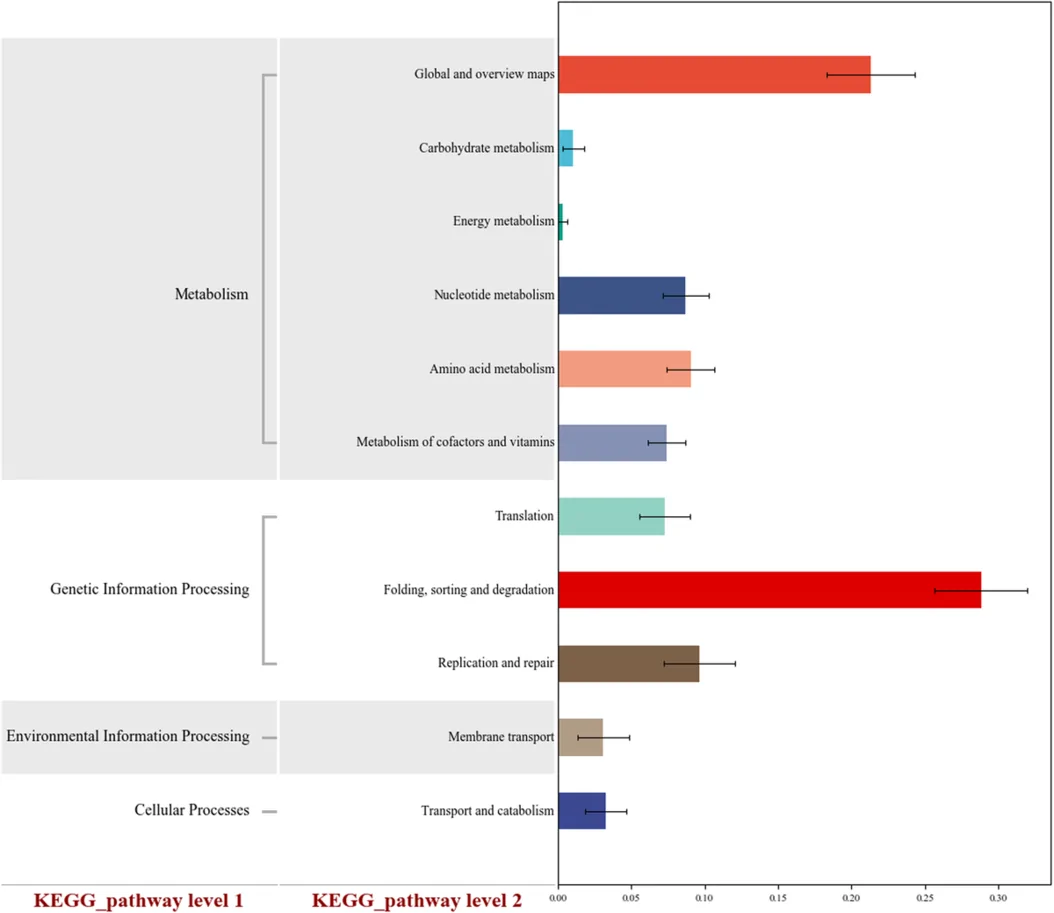
KEGG pathway functional profiles of microbial communities (level 1/2). Bars show relative abundance; error bars denote SD.

KEGG pathway enrichment analysis of functional genes derived from the differential microbiota revealed a predominance of metabolism‐related pathways Figure 8. Among level‐1 KEGG pathways, genes involved in metabolic processes exhibited the highest abundance, highlighting their potential contribution to tumor‐associated microbial function.

### 3.10 *Malassezia* Promotes Cell Proliferation in HaCaT and A‐431 Epidermoid Carcinoma Cells

#### 3.10.1 CCK‐8 Assay

**Figure 9:**
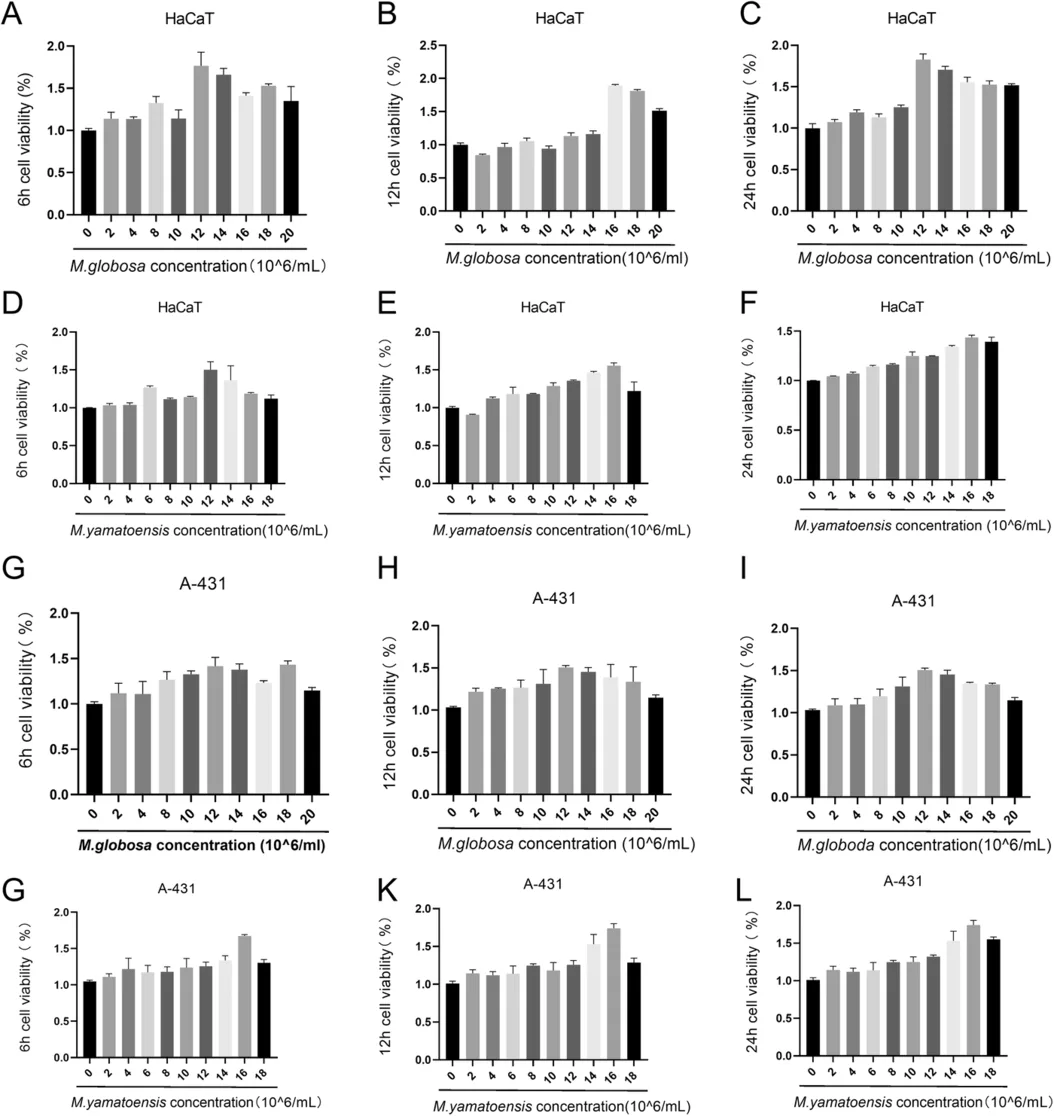
Changes in cell viability (%) of HaCaT cells (immortalized keratinocytes) and A‐431 cells (epidermoid carcinoma cell line used as surrogate skin malignant model) after treatment with different concentrations of *M. globosa and M. yamatoensis* for 6, 12, and 24 h. Panels A–C and G–I show results for HaCaT cells; panels D–F and J–L show results for A‐431 cells. Panels A, D, G, and J correspond to 6‐h B, E, H, and K to 12‐h; panels C, F, I, and L to 24‐h.

The proliferative effects of different concentrations and time points of *M. globosa* and *M. yamatoensis* on HaCaT and A‐431 epidermoid carcinoma cells were assessed using a CCK‐8 assay (Figure 9). HaCaT and A‐431 epidermoid carcinoma cell proliferation exhibited a biphasic pattern, initially increasing and then decreasing, with variations in response to various concentrations of *M. globosa* and *M. yamatoensis* at 6, 12, and 24 h. The most pronounced proliferative response was observed at 12 h with *M. globosa* (1.2 × 10^7^ CFU/mL) and *M. yamatoensis* (1.6 × 10^7^ CFU/mL).

#### 3.10.2 EDU Assay

**Figure 10:**
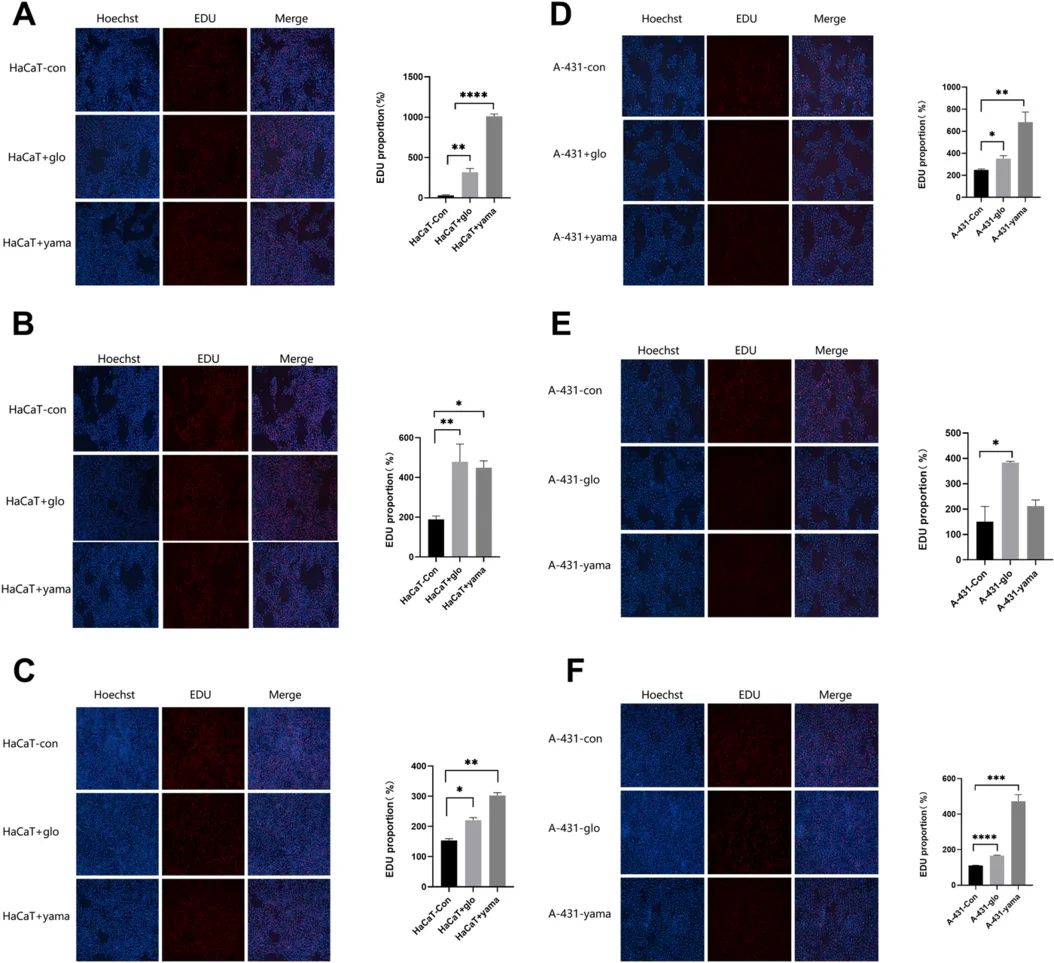
EdU fluorescence staining and quantitative analysis showing the proportion of EdU‐positive cells (reflecting cell proliferation) in HaCaT cells (A–C) and A‐431 cells (epidermoid carcinoma cell line used as surrogate skin malignant model) (D–F) treatment with *M. globosa* and *M. yamatoensis* for 6, 12, and 24 (A, D), 6‐h treatment; (B, E), 12‐h treatment; (C, F), 24‐h treatment. For each panel, the left side shows representative fluorescence images: Hoechst (nuclei, blue), EdU (proliferating cells, red), and merged channels. The right side presents corresponding bar graphs quantifying the percentage of EdU‐positive cells. Significance between groups is indicated as follows: *p < 0.05, **p < 0.01, ***p < 0.001,****p < 0.0001.

EdU incorporation confirmed the pro‐proliferative effect of *Malassezia* (Figure 10D–F). At 6 h (Figure 10), both *M. globosa* and *M. yamatoensis* significantly increased the proportion of EdU‐positive cells compared with the control group (*p* < 0.05, *p < 0.01), with *M. yamatoensis* exhibiting a more pronounced effect. At 12 h of treatment (Figure 10E), *M. globosa* showed a significant increase in percentage of EdU‐positive cells compared to the control group (*p* < 0.05), whereas *M. yamatoensis* did not differ significantly from the control. By 24 h (Figure 10F), both *M. globosa* and *M. yamatoensis* significantly elevated EdU EdU‐positive cell proportions compared to the control (**p < 0.001, ***p < 0.0001), with *M. yamatoensis* again showing a more significant increase.

### 3.11 Transcriptomic Profiling and PCA Analysis Between Groups

**Figure 11:**
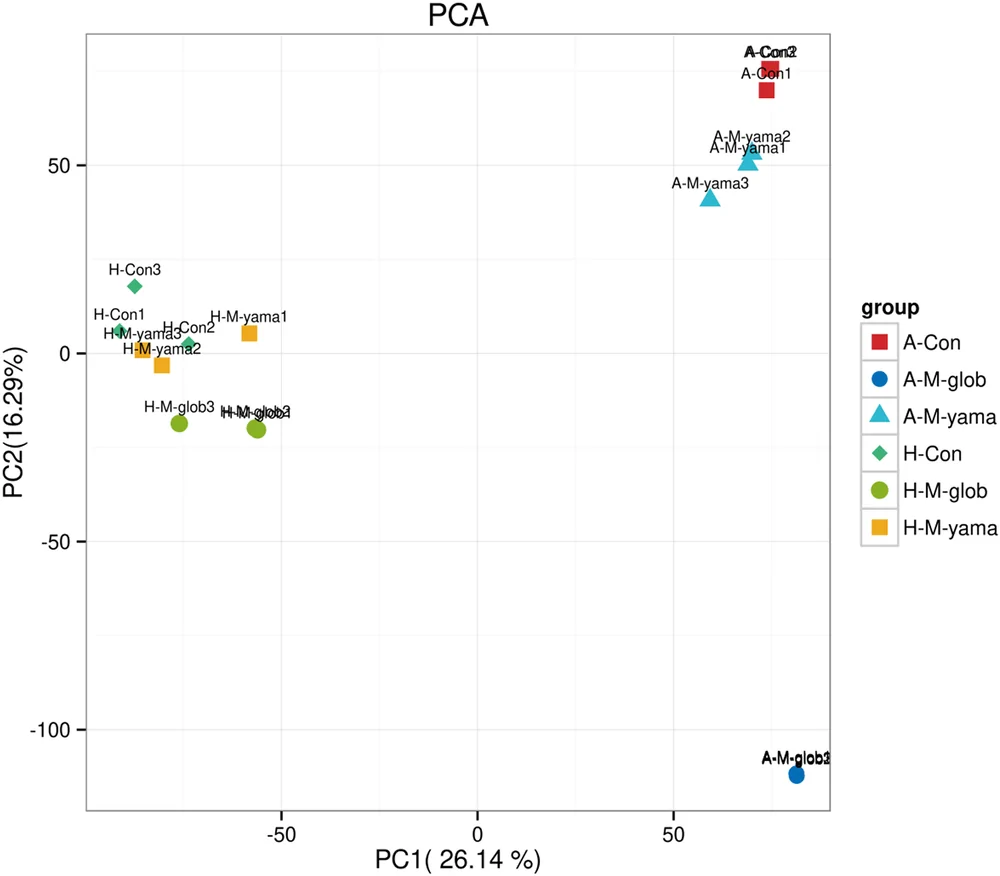
Principal component analysis of transcriptome profiles. Groups: A‐Con (untreated A‐431 cells, epidermoid carcinoma cell line used as surrogate skin malignant model), A‐M‐glob (*M. globosa*‐treated A‐431), A‐M‐yama (*M. yamatoensis*‐treated A‐431); H‐Con/H‐M‐glob/H‐M‐yama correspond to untreated and fungus‐treated normal HaCaT keratinocytes. Clear inter‐group separation and tight intra‐group clustering indicate reliable transcriptomic data.

PCA demonstrated clear and independent distribution between transcriptomic profiles of HaCaT and A‐431 epidermoid carcinoma cells cocultured with *M. globosa* and *M. yamatoensis* (Figure 11). Distinct inter‐group separation and high intra‐group clustering were observed, confirming robust biological reproducibility and strong data reliability.

### 3.12 Volcano Plot Identification of DEGs

**Figure 12:**
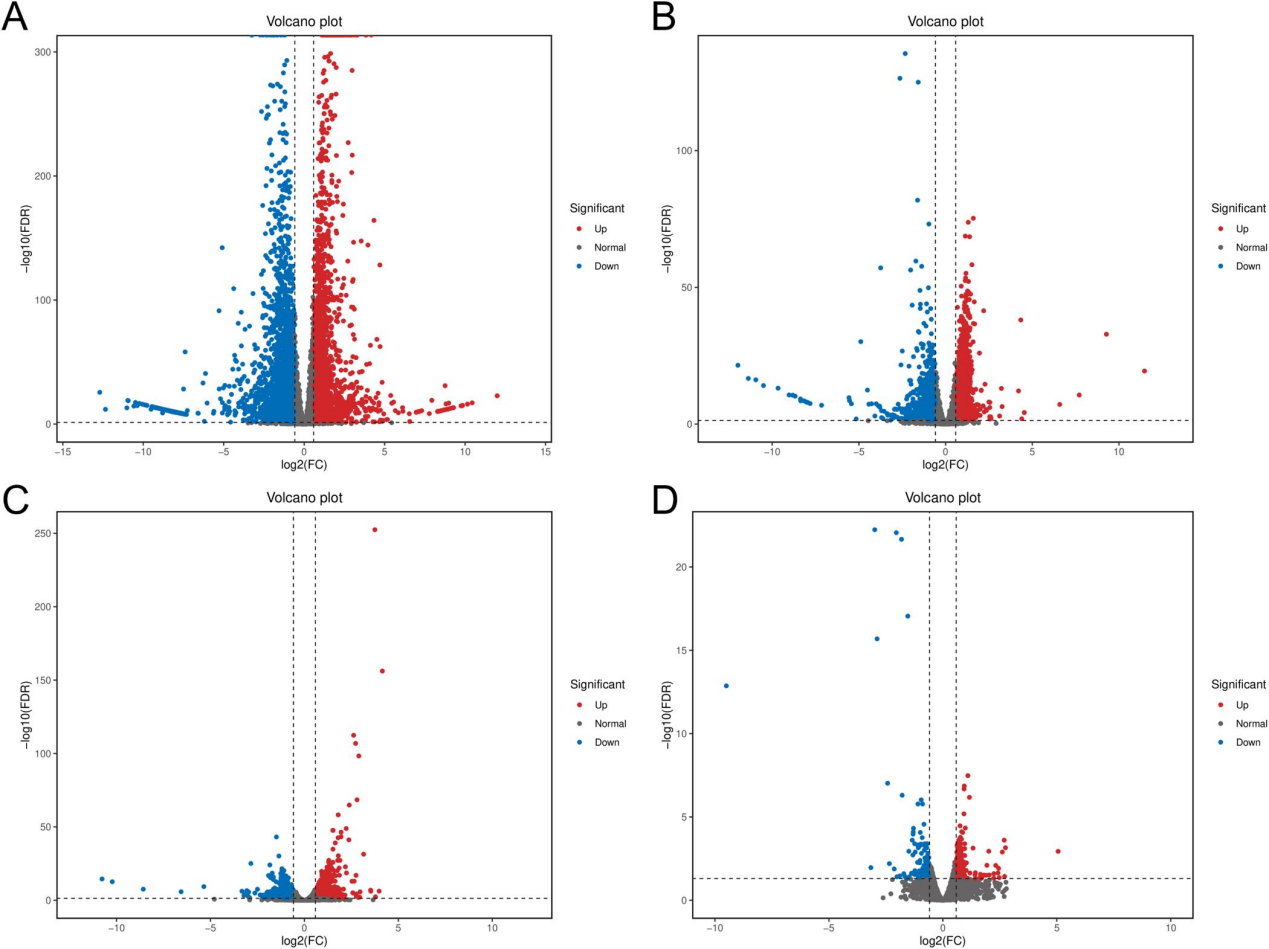
Volcano plots of differentially expressed genes. (A, B) A‐431 cells (epidermoid carcinoma cell line used as surrogate skin malignant model) with/without *M. globosa*/*M. yamatoensis stimulation*; (C, D) HaCaT keratinocytes with/without fungal stimulation. Red dots: significantly upregulated genes; blue dots: significantly downregulated genes; gray dots: non‐differentially expressed genes.

As illustrated in Figure 12, stimulation with *M. globosa* for 12 h induced 1845 DEGs in HaCaT cells, of which 903 were upregulated and 942 were downregulated. *M. yamatoensis* treatment yielded 530 DEGs, including 319 upregulated and 211 downregulated genes. In A‐431epidermoid carcinoma cells, *M. globosa* exposure resulted in 5727 DEGs (2,674 upregulated and 3053 downregulated), whereas *M. yamatoensis* stimulation led to 3247 DEGs, comprising 1794 upregulated and 1453 downregulated genes (adjusted *p* < 0.05, |log_2_ fold change | > 1). These findings highlight strain‐ and cell line‐specific transcriptomic responses to *Malassezia*.

### 3.13 KEGG Pathway Enrichment Analysis

**Figure 13:**
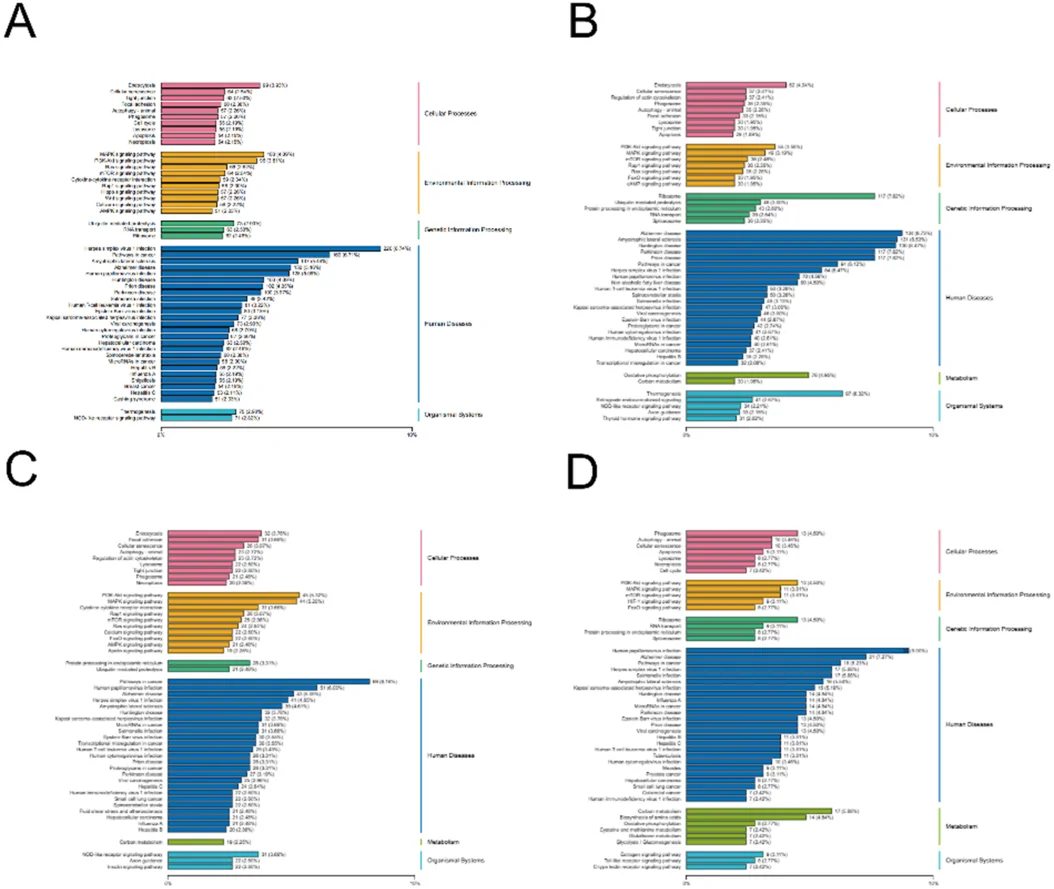
KEGG pathway enrichment analysis of DEGs. (A,B): A‐431 cells (epidermoid carcinoma cell line used as surrogate skin malignant model) stimulated by *M. globosa* and *M. yamatoensis*, respectively; (C,D): corresponding results from HaCaT keratinocytes.

As shown in Figure 13, the KEGG pathway enrichment analysis revealed that stimulation of A‐431 cells with both *M. globosa* and *M. yamatoensis* significantly enriched key pathways associated with inflammation and oxidative stress. In terms of inflammatory response, both strains commonly enriched the NOD‐like receptor signaling pathway and the MAPK signaling pathway. Additionally, *M. globosa* specifically enriched the Cytokine‐cytokine receptor interaction pathway, while *M. yamatoensis* specifically enriched the PI3K‐Akt signaling pathway. Regarding oxidative stress, both strains consistently induced significant enrichment in oxidative phosphorylation.

### 3.14 Effects of *Malassezia* on Inflammatory and Oxidative Stress Gene Expression

**Figure 14:**
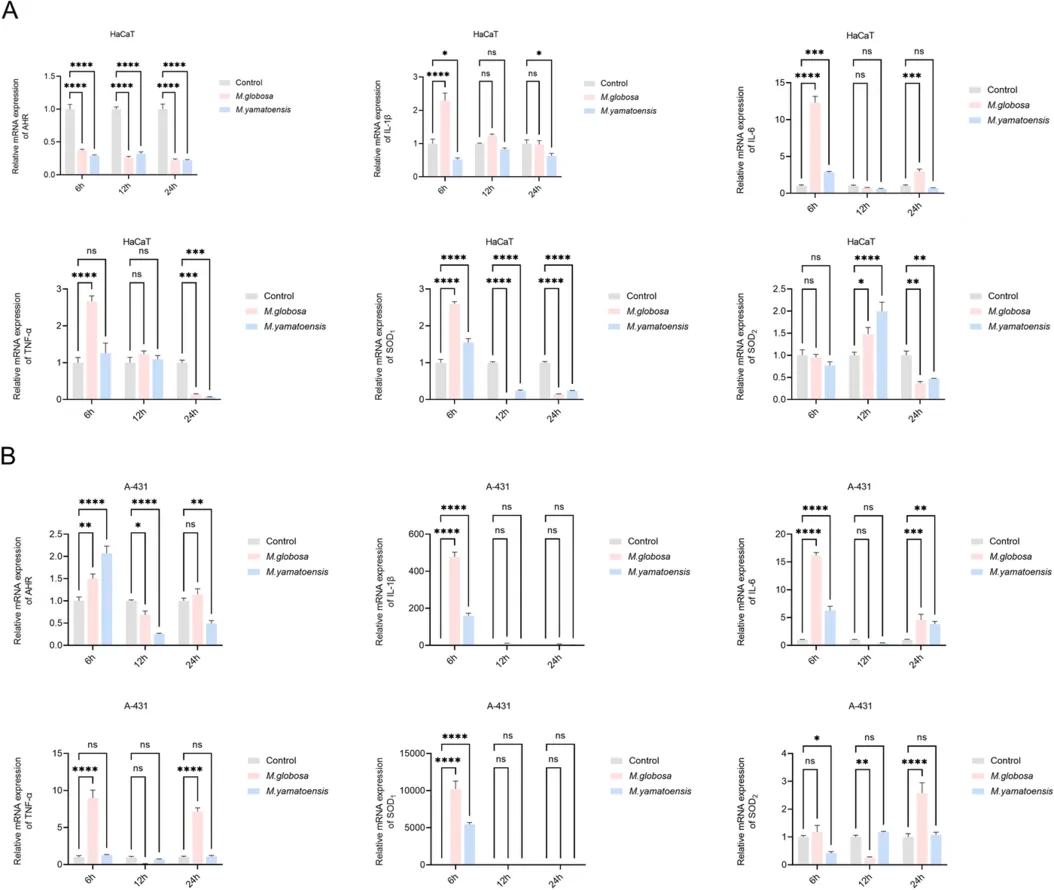
Relative mRNA expression of inflammatory and oxidative stress‐related genes detected by RT‐qPCR. (A) Normal HaCaT keratinocytes; (B) A‐431 cells (epidermoid carcinoma cell line used as surrogate skin malignant model) co‐cultured with *M. globosa/M. yamatoensis* for 6, 12, 24 h. Data shown as mean ± SD; ns = not significant, *p < 0.05, **p < 0.01, ***p < 0.001, ****p < 0.0001.

RT‐qPCR analysis validated the transcriptomic findings by quantifying the expression of pro‐inflammatory cytokines (*IL‐1β*, *IL‐6*, and *TNF‐α*) and oxidative stress‐related genes (*SOD*_*1*_ and *SOD*_*2*_) in HaCaT and A‐431 epidermoid carcinoma cells exposed to *M. globosa* and *M. yamatoensis* for 6, 12, and 24 h (Figure 14). *M. globosa* induced significantly stronger responses than *M. yamatoensis* in both cell lines, with IL‐1β showing sustained upregulation at all time points (*p* < 0.0001), *TNF‐α* and IL‐6 exhibiting peak elevation at 6 h (*p* < 0.0001), and *SOD*_*1*_ demonstrating robust increases across all time points (*p* < 0.0001), while *SOD*_*2*_ expression was significantly elevated primarily at 12 h and 24 h (*p* < 0.01 to *p* < 0.0001).

## 4 Discussion

Our clinical data revealed no significant gender disparities in BCC incidence, and the highest prevalence was found in patients aged 60–70 years. These epidemiological characteristics are consistent with previous reports stating that BCC predominantly affects elderly populations (Ciuciulete et al. 2022). Globally, the incidence of non‐melanoma skin cancers has risen sharply among adults over 60 years since 1990, with BCC cases increasing by 291.85% (Pan et al. 2025). Advanced age correlates with cumulative ultraviolet (UV) exposure, intrinsic skin aging, impaired DNA repair and weakened cutaneous immunity, all of which collectively elevate BCC risk (Halilovic et al. 2019). Our results are consistent with domestic and international cohort findings (Valian et al. 2024). In our cohort, most lesions occurred on the head and face, especially the nasal and periorbital areas, which further supports the well‐established role of chronic UV exposure as a major pathogenic factor of BCC (Czaplicka et al. 2022). Histopathologically, nodular BCC was the dominant subtype, in line with the findings of prior clinical studies (McDaniel and Steele 2025). BCC generally progresses slowly and presents low invasiveness, as reflected by the median disease duration of 3 years in our patients. From a public health perspective, targeted screening, sun protection education and early intervention strategies should be prioritized for elderly high‐risk groups. That said, our epidemiological cohort bears inherent limitations: the sample size is modest, and only histologically verified BCC patients were enrolled. Follow‐up large‐scale cohorts incorporating diverse skin lesions are needed to corroborate these clinical findings.

Fungi represent core components of the human microbiota, and accumulating evidence suggests their potential involvement in tumor initiation, progression and clinical prognosis (Narunsky‐Haziza et al. 2022). A growing body of evidence also shows that fungal communities are closely linked to cancer development, patient prognosis and treatment responses (Cheng et al. 2024). Distinct fungal community profiles have been identified in various tumor tissues, suggesting potential functional roles for fungi across different cancer types. *Malassezia*, a dominant lipophilic fungus colonizing human skin, has been detected at high levels in pancreatic, breast, liver and colorectal cancers (Aykut et al. 2019). However, while *Malassezia* species have recently been detected in BCC lesions, their functional relevance to BCC pathogenesis remains largely unexplored (Koç Yıldırım et al. 2024). Previous studies on skin tumors have mainly focused on bacterial pathogens. For instance, higher colonization rates of *Staphylococcus aureus* have been observed in cutaneous squamous cell carcinoma (SCC) lesions compared with healthy skin, and cutaneous microbial dysbiosis is hypothesized to facilitate malignant transformation of skin lesions (Lewis et al. 2019). Similarly, our sequencing data revealed multiple *Malassezia* species colonizing BCC tissues, among which *M. restricta* and *M. globosa* were predominant. Multiple *Malassezia* species with high relative abundance were detected in both cancerous tissues (CT) and peritumoral tissues (PT) across our limited three paired fresh tissue samples, showing a tentative consistency with the known mycobiome composition of healthy human skin (Park et al. 2021). This observation remains preliminary and requires validation in expanded fresh tissue cohorts.

Prior cutaneous *Malassezia* studies largely concentrate on superficial inflammatory skin conditions; while one early report hypothesized its involvement in skin carcinogenesis (Gaitanis et al. 2011), and a later molecular survey detected *Malassezia* species in BCC lesions (Koç Yıldırım et al. 2024), neither investigation performed functional experiments to validate its tumor‐modulating capacity. In this in vitro study, we employed two complementary skin‐derived epithelial models—HaCaT (immortalized keratinocytes) and A‐431 (epidermoid carcinoma) —and verified that both *M. globosa* and *M. yamatoensis* can significantly accelerate the proliferation of both cell lines. We note that A‐431 is an epidermoid carcinoma line, not a BCC line; thus, these in vitro findings should be interpreted as evidence that *Malassezia* can stimulate epithelial proliferation in broadly relevant keratinocyte/carcinoma models, rather than as BCC‐specific proof. Such growth‐promoting phenotypes may be mediated by fungal metabolites. Consistent with our findings, studies have shown that *M. globosa* is enriched in pancreatic tumors and promotes their growth through the MBL–C3 complement pathway (Aykut et al. 2019). Similarly, colonization by *M. globosa* has been reported to upregulate IL‐17A expression, drive macrophage polarization toward the M2 phenotype, and correlate with accelerated breast cancer progression (Liu et al. 2024). Mechanistically, *Malassezia* can secrete free fatty acids to disrupt epithelial barriers, induce pro‐inflammatory cytokine release and degrade the ECM, which may jointly shape a microenvironment permissive to tumor cell growth. The full signaling cascades underlying these phenotypes remain to be clarified and warrant further mechanistic dissection.

Notably, *M. yamatoensis* displayed divergent abundance patterns between CT and PT, with higher abundance in peritumoral tissues. Although its overall abundance was lower than *M. globosa*, our cellular assays demonstrated that *M. yamatoensis* stimulation induced proliferative and inflammatory responses comparable to *M. globosa*. This indicates that microbial functional effects within the tumor microenvironment cannot be simply evaluated based on relative abundance; even low‐abundance species may exert profound biological impacts via bioactive metabolites and fungus‐host molecular crosstalk (Poore et al. 2020). It should be noted that higher *Malassezia* abundance in PT relative to CT does not necessarily indicate a driving role in tumor progression. The lipid‐rich peritumoral niche may simply favor *Malassezia* colonization, meaning that higher PT abundance could be a consequence of the altered tissue landscape rather than a cause. PT samples may also inadvertently include adnexal structures such as hair follicles and sebaceous glands that naturally harbor fungi, which would introduce sampling bias. Another possibility is that tumor formation and tissue remodeling reshape local skin microecology—tumor cores restrict fungal survival through hypoxia, nutrient scarcity, and antimicrobial peptide release, so PT fungal enrichment may simply reflect tumor‐induced niche changes. Nevertheless, the consistent enrichment of *Malassezia* in peritumoral tissue does suggest a relationship between this fungus and the BCC microenvironment, the nature of which requires further investigation. Notably, no extraction blanks or no‐input sequencing controls were included in our metagenomic workflow, owing to the limited number of fresh tissue samples. For low‐biomass skin specimens, reagent‐ or environment‐derived contamination cannot be fully excluded, and the abundance data of rare fungal taxa should therefore be interpreted tentatively. Future studies incorporating comprehensive negative controls and larger patient cohorts are warranted to validate the differential distribution of *Malassezia* between tumor and peritumoral tissues.

At the transcriptional level, our KEGG enrichment analysis inferred the potential involvement of several signaling pathways, including NOD‐like receptor, MAPK, PI3K‐Akt, and oxidative phosphorylation. While these cell‐line‐derived transcriptional signatures suggest that *Malassezia* may engage inflammatory and oxidative stress programs, they do not constitute functional validation of these signaling axes in BCC tissue. Inflammation and oxidative stress are two core pathways driving tumor development. The fungal cell wall component β‐glucan serves as a pattern recognition ligand to activate the NLRP3 inflammasome (Kankkunen et al. 2010). Pathogenic fungi such as *Candida albicans* and *Aspergillus fumigatus* trigger IL‐1β secretion, thereby establishing a chronic inflammatory niche that favors carcinogenesis (Gross et al. 2009; Joly et al. 2009; Zhang et al. 2022). Analogously, *Malassezia* can also activate the NLRP3 inflammasome via the Dectin‐2/CARD9 signaling cascade to amplify local inflammatory responses (Douglas et al. 2015; Tsang et al. 2021). Persistent inflammation induces excessive reactive oxygen species (ROS) production, which causes DNA damage and accelerates tumorigenesis (Fernandes et al. 2024). ROS not only directly compromises genomic stability but also facilitates cancer cell proliferation and tumorigenesis by activating redox‐dependent signaling pathways (Canli et al. 2017). In skin tissues, *Malassezia* can secrete toxic proteases and stimulate host cells to generate ROS, aggravating local inflammatory injury (Später et al. 2009). As a key intracellular antioxidant enzyme, superoxide dismutase (SOD) plays a central role in scavenging ROS and defending against oxidative stress (Zhao et al. 2021; Wang et al. 2018). Increased SOD activity represents a compensatory response to oxidative stress, and the dynamic balance between ROS and SOD plays a critical role in regulating cell biological behaviors (Li et al. 2021). This redox feedback mechanism observed in prior studies matches the molecular phenotypes detected in our *Malassezia*‐treated cell models, providing a feasible framework to interpret fungus‐mediated oxidative stress in human skin.

Inflammatory cytokines and oxidative stress markers have emerged as auxiliary candidate indicators for tumor diagnosis. Elevated levels of cytokines, such as IL‐1β, IL‐6, and TNF‐α, have been reported in breast CT compared to PT (Lan et al. 2021), where they regulate the replication of cancer stem cells and contribute to malignant growth (Wang et al. 2020). Upregulated IL‐1β, IL‐6 and TNF‐α have been widely documented across multiple carcinoma types and correlate with enhanced malignant cell proliferation (Xia 1998). A bidirectional regulatory relationship exists between inflammation and oxidative stress, and both pathways represent promising therapeutic targets for oncology treatment (Kotsafti et al. 2020). SOD are major cellular antioxidants with context‐dependent pro‐tumor activities (Liu et al. 2020). Studies have confirmed the oncogenic role of SOD1 in multiple malignancies, and targeted inhibition of SOD1 can induce cancer cell apoptosis (Glasauer et al. 2014). In the present study, transcriptomic analysis and RT‐qPCR validation revealed that *Malassezia* stimulation significantly upregulated *IL‐1β*, *IL‐6*, *TNF‐α*, *SOD1,* and *SOD2* in A‐431 and HaCaT cells. Elevated pro‐inflammatory cytokines indicated sustained activation of inflammatory pathways, while increased *SOD* expression reflected a compensatory oxidative stress response. Combined, these molecular alterations may support the proliferative activity of skin‐derived epithelial cells, and the inflammatory and oxidative stress programs identified here overlap with pathways implicated in several epithelial tumor types. Our findings suggest the crosstalk between *Malassezia*‐mediated inflammation and oxidative stress in these epithelial models, and supply new in vitro experimental evidence linking skin fungi to molecular phenotypes relevant to, but not specific to, BCC.

In summary, our sequencing data reveal an association between cutaneous *Malassezia* and BCC, with *M. globosa* as the predominant species. Higher *Malassezia* abundance in PT relative to CT does not imply a causal role and may instead reflect microenvironmental selection, sampling bias, or reverse causation. As a microbial factor modulating epithelial inflammation and oxidative stress, *Malassezia* may represent a tentative candidate biomarker and auxiliary therapeutic target for BCC; comprehensive preclinical and clinical validation is required to verify its translational potential.

## 5 Conclusion

In this study, *Malassezia* distribution and abundance in BCC were characterized with two complementary approaches: species‐specific RT‐qPCR screening of 50 archived paraffin‐embedded BCC specimens and metagenomic sequencing of three pairs of fresh tumor and peritumoral tissues. Subsequent in vitro cell experiments, using HaCaT keratinocytes and A‐431 epidermoid carcinoma cells as complementary skin‐derived epithelial models, showed that both *M. globosa* and *M. yamatoensis* markedly enhance the proliferation of both cell lines. Transcriptome analysis combined with RT‐qPCR further indicated that the pro‐proliferative activity of *Malassezia* was associated with altered inflammatory and oxidative stress pathways. The expression of pro‐inflammatory cytokines (*IL‐1β*, *IL‐6* and *TNF‐α*) and oxidative stress‐related genes (*SOD1* and *SOD2*) was significantly upregulated. Sustained elevation of *IL‐1β* indicates persistent pro‐inflammatory responses in these cell models, while increased *SOD1* expression reflects a compensatory oxidative stress response.

Collectively, our in vitro results suggest that *Malassezia* can modulate inflammatory and oxidative stress signaling in association with enhanced proliferation of skin‐derived epithelial cells. While these cellular models (HaCaT and A‐431) are not BCC‐specific, the identified pro‐proliferative and pro‐inflammatory phenotypes suggest that *Malassezia* colonization could, in principle, contribute to a microenvironment permissive to epithelial tumor growth—a hypothesis that remains to be tested in BCC‐specific models. This study provides novel in vitro evidence linking skin commensal *Malassezia* to epithelial proliferative phenotypes relevant to BCC, and points to this fungus as a tentative biomarker requiring rigorous preclinical and clinical validation.

### 5.1 Limitations

Although this study provides in vitro evidence from skin‐derived epithelial models, several methodological limitations need to be addressed.

First, FFPE tissues used for fungal molecular analysis have inherent technical limitations. Formalin fixation, paraffin embedding, and prolonged storage all degrade DNA integrity, which partially affects the sensitivity and specificity of nucleic acid‐based detection. Because our paraffin blocks had been archived for many years, the original embedding and dewaxing reagents were no longer available, so matched blank extraction negative controls could not be prepared for qPCR. These issues are common in retrospective FFPE‐based studies, however, and do not invalidate fungal load comparisons within our cohort. We also did not have enough matched fresh tissues to perform systematic culture‐based validation. Although frozen specimens and culture methods generally provide more accurate microbial identification, *Malassezia* are fastidious and difficult to culture in vitro, and cross‐contamination may also skew species‐level results from FFPE sections. Future studies incorporating culture validation and more complete clinical metadata will help confirm these findings.

Second, metagenomic sequencing was limited by a small fresh tissue cohort (*n* = 3 pairs) and the absence of extraction blanks or no‐input library controls. The modest sample size restricts generalizability and likely explains some discrepancy between the 50 FFPE qPCR results and the metagenomic species profiles. More critically, skin tissue has extremely low fungal biomass against a heavy human DNA background; without blank controls, we cannot rule out reagent or environmental contamination for low‐abundance taxa. Consequently, the relative abundance of rare *Malassezia* and minor genera shown in Figure 7 should be treated as tentative. Similarly, the apparent PCA‐based separation between CT and PT fungal communities (Figure 6) should be interpreted with caution, as it is based on only three paired samples. Future work should employ larger sample sizes, parallel negative controls, and orthogonal methods such as qPCR or FISH to validate these preliminary observations.

Third, we only enrolled patients with pathologically confirmed BCC and excluded other skin lesions with overlapping risk factors. The limited clinical sample size and narrow inclusion scope may introduce potential bias.

Fourth, while our in vitro assays indicated that *Malassezia* exposure may boost cell proliferation and activate inflammatory signaling pathways, the complete molecular mechanisms underlying these cellular phenotypes remain incompletely defined. Previous studies hypothesize that *Malassezia* may exert biological activities via the AHR signaling pathway; however, further experimental work is required to verify whether this pathway mediates the proliferative cell responses triggered by *Malassezia*. There are also inherent limitations in that the fungal concentrations and brief co‐culture durations employed in this in vitro study cannot fully recapitulate the actual in vivo exposure conditions.

Fifth, the in vitro experiments were conducted using HaCaT keratinocytes and A‐431 epidermoid carcinoma cells. A‐431 is an epidermoid carcinoma line, not a BCC line, and HaCaT cells are immortalized but non‐transformed keratinocytes. Thus, while these cell lines serve as broadly relevant epithelial models for evaluating *Malassezia*‐induced proliferative and inflammatory responses, they do not recapitulate the specific biology of BCC. Future studies should validate these findings in BCC‐derived cell lines or patient‐derived BCC organoids to confirm that the observed phenotypes are relevant to BCC specifically.

Notably, the present work only identifies an associative relationship between cutaneous *Malassezia* colonization and BCC, together with supportive in vitro cellular data. Causal evidence confirming that *Malassezia* drives BCC progression is lacking in our current dataset. Large prospective cohorts and in vivo animal models are warranted to further explore its potential pathogenic relevance and translational value as a biomarker or therapeutic target.

## Author Contributions

**Xiaolei Zhu:** software; writing – original draf, formal analysis, data curation, validation. **Wen Zhang:** conceptualization, methodology, formal analysis, data curation, supervision, writing – review and editing. **Tinghong Ye:** conceptualization, methodology, software, validation, writing – review and editing. **Heng Zhang:** conceptualization, supervision, funding acquisition, project administration, writing – review and editing. **Jufang Tan:** methodology, conceptualization, funding acquisition, resources, writing – review and editing. **Yi Sun:** conceptualization, methodology, data curation, formal analysis, supervision, funding acquisition, resources, project administration, writing – review and editing.

## Ethics Statement

The experimental protocol was reviewed and approved by the Ethics Committee of Jingzhou Hospital Affiliated with Yangtze University (Approval No.: 2024‐187‐01). Informed consent was obtained from all individual participants included in the study.

## Consent

The authors have nothing to report.

## Conflicts of Interest

The authors declare no conflicts of interest.

## 1 Data Availability Statement

The data that support the findings of this study are openly available in NCBI SRA at https://www.ncbi.nlm.nih.gov/bioproject, reference numbers PRJNA1243595 PRJNA1418325. The metagenomic sequencing and RNA‐seq transcriptomic datasets generated during the current study have been deposited in the NCBI Sequence Read Archive (SRA) repository and are publicly accessible under the following accession numbers: [accession number: SRR32909515,SRR32909516; Bio Project ID: PRJNA1243595] for metagenomic data and [accession number: SRR37094783,SRR37094784,SRR37094785,SRR37094786,SRR37094787,SRR37094788; Bio Project ID: PRJNA1418325] for RNA‐seq data.
